# Dosages of Swallowing Exercises Prescribed in Stroke Rehabilitation: A Medical Record Audit

**DOI:** 10.1007/s00455-022-10500-x

**Published:** 2022-08-11

**Authors:** Jacinda Choy, Fereshteh Pourkazemi, Caitlin Anderson, Hans Bogaardt

**Affiliations:** 1 Faculty of Medicine and Health, Sydney School of Health Sciences, The University of Sydney, Sydney, NSW 2006 Australia; 2 HammondCare Braeside Hospital, 340 Prairie Vale Road, Prairiewood, NSW 2176 Australia; 3 School of Allied Health Science and Practice, University of Adelaide, Adelaide, SA 5005 Australia

**Keywords:** Stroke, Intervention, Dysphagia, Exercise, Dose, Medical Records

## Abstract

This study investigated how swallowing exercise dosage is recorded, and what swallowing exercise dosages are reported in a stroke rehabilitation setting. We additionally explored the relation between mean daily swallowing repetitions and likelihood of improvement in functional swallowing status and considered how swallowing exercise dosages in practice compared to evidence-based principles of neural plasticity and strength training. We audited medical records for 42 patients with post-stroke dysphagia admitted to an inpatient rehabilitation unit over 18 months. Data were collected on participant characteristics, swallowing exercises and dosages, and clinical outcomes. The relation between dosage and outcomes was investigated using logistic regression analysis. On average, patients were seen for a median of 2.4 swallowing intervention sessions per week (IQR: 1.7) over 21 days (IQR: 16) and received a median 44.5 swallowing exercise repetitions per session (IQR: 39.6). Results indicated variable reporting of swallowing exercise dosages. Frequency, intervention duration, exercise type, and number of repetitions were routinely recorded in medical records, while intensity, session length, content, and adherence to home exercise programs were not. Frequency of swallowing intervention was lower in practice compared to research studies, and swallowing exercises did not follow specificity or progressive resistance principles. Likelihood of improvement in swallowing status was partially explained by age (B = -.015, *p* = .007) but not by mean daily swallowing exercise repetitions. This study illustrates dosages of swallowing exercises used in clinical practice. Results highlight the need for improved consideration and reporting of dosage, and application of evidence-based principles to swallowing exercise dosages.

## Background

Dosage is a key aspect of exercise prescription for all populations with most research relating to gross motor exercise. Exercise is defined as any structured physical action to improve fitness or health [1, 2]. Exercise dosage consists of four components: frequency (how often), intensity (effort level), time (duration), and type (what mode) of exercise – known as the FITT principle [3]. A dose–response relationship has been demonstrated, with increased exercise dosage associated with reduced mortality [4, 5], improved quality of life [6], and muscle hypertrophy [7]. This relationship between exercise dosage and outcomes has been demonstrated in a range of populations, including healthy adults [8], older adults [9–11], and adults with diseases [12] including stroke populations [13, 14]. The emphasis on the importance of exercise dosage is shown by the wealth of research into dosage recommendations for gross motor exercise for different populations [2, 3, 15–17].

In stroke rehabilitation, dosage, particularly amount of intervention, is linked to experience-dependent neural plasticity. The principles of neural plasticity identify factors which maximize the ability of the central nervous system to adapt in response to experience [18–20]. A key dosage-related principle of neural plasticity is amount of intervention. Increased amount of intervention facilitates structural brain changes, neural reorganization, and improved motor limb function after stroke [18, 21]. Increasing the amount of intervention (whether number of repetitions, minutes of active intervention, or scheduled session time) results in improved outcomes in limb rehabilitation after stroke [22, 23]. Meta-analyses of randomized controlled trials demonstrate that additional physical intervention after stroke is associated with improved activities of daily living [24], therapy outcomes [25], and upper and lower limb activity [26]. Thus, amount of exercise impacts on intervention outcomes in stroke populations.

Another principle of neural plasticity is specificity, where type of training influences type of motor learning [18, 20]. Task-specific or task-oriented practice – where the trained task matches a functional task – has been shown to be effective in stroke rehabilitation [27–30]. Intervention studies demonstrate positive outcomes with upper limb and mobility task-oriented training compared to traditional non-task-specific intervention. For example, Van de Port [31] found improved gait speed, walking distance, and stair mobility in a task-oriented circuit training group compared with usual individual physiotherapy in adults after stroke. Thant et al. [28] conducted a small randomized controlled trial (RCT) which found significant improvements in upper limb motor ability, speed, and in patient-reported use of the affected hand. Systematic reviews exploring the combined effects of RCTs show that task-oriented training in upper and lower limb stroke rehabilitation is more effective than traditional therapy in improving motor function, functional activity, and health-related quality of life [27, 30, 32].

Another consideration in intervention dosage for stroke patients is strength training. Muscle weakness – resulting from reduced neural activation and muscle atrophy post-stroke – is strongly associated with reduced function and independence [33]. Thus, strength training is an important adjunct to functional intervention to reverse post-stroke weakness [16, 33]. Strength training principles include progressive overload – gradual increase of exercise demands, and resistance training – training the muscle against an external load to build muscle bulk and strength [34, 35]. Intervention studies demonstrate positive outcomes of progressive resistance training incorporated with other exercises. For example, Vahlberg et al. [36] found that progressive resistance and balance exercises combined with motivational discussions improved balance and walking speed in adults with chronic stroke. Yang [37] found that task-oriented progressive resistance training improved lower limb strength and functional measures, including gait velocity, cadence, and stride length. Overall, dosage, particularly amount of intervention, and exercise type – including task-specific and strength-based training – impact on outcomes of stroke intervention. However, these effects have primarily been investigated in upper and lower limb training.

Exercise is also used to rehabilitate impaired swallowing after stroke. Swallowing exercises capitalize on neural plasticity to invoke long-term improvement in swallowing function [38, 39]. Swallowing exercises can be categorized into indirect swallowing exercises (motor without swallow) which target swallowing-related muscles and direct swallowing exercises (motor with swallow) which involve swallowing [40, 41]. An alternative way to categorize swallowing intervention is into strength-based exercises which aim to strengthen oral and pharyngeal muscles and skill-based exercises which focus on refinement of impaired swallowing biomechanics [34, 42]. Similar to limb exercises in stroke rehabilitation, swallowing exercises aim to improve muscle strength and skilled task performance. Swallowing differs from limb function due to the involvement of reflexive and volitional components and use of specialized oral and pharyngeal muscles [43]. However, it is likely that exercise principles of neural plasticity apply to swallowing, given early evidence of cortical changes and motor adaptation in swallowing [44]. Research has demonstrated improvement in level of oral intake, tongue strength, and swallowing-related quality of life, and reduction in aspiration and medical complications when applying exercise principles to swallowing intervention, for example, when increasing intervention intensity [45, 46] or using resistance-based exercises [47, 48]. As a result, experts in the field postulate the importance of using neural plasticity principles to guide dosage of dysphagia rehabilitation [34, 39, 40].

However, dosage of swallowing exercises is relatively less explored than dosage of limb exercises. Despite evidence on the importance of exercise dosage, literature reviews show inconsistent reporting of dosage in intervention studies on swallowing exercises after stroke [49, 50]. Similarly, surveys of speech pathologists reveal inconsistent and varied prescription of swallowing exercise dosages [51, 52]. A wide range of different swallowing exercise types, inconsistency in dosage definition and reporting, and variation in study populations and outcome measures results in a paucity of clear evidence-based dosage guidelines for swallowing exercises [49]. Further, an ongoing knowledge–action gap due to service delivery factors, patient factors, and limitations on clinically applicable research contributes to difficulty understanding how to apply evidence on principles of dosage into clinical practice [53].

Therefore, it is unclear whether and how evidence on neural plasticity and dosage is used when prescribing swallowing exercise dosages in clinical practice. Given the lack of clarity in the literature, this study aims to investigate the question from a clinical perspective. In order to understand current clinical practice in dosage recording, prescription and implementation, and determine a baseline from which to improve practice, more information is needed on the type and dosages of swallowing exercises used in clinical settings. There has been no recently published audit investigating the dosages of swallowing exercises recorded and used in a clinical setting. Therefore, this study was conducted to compare clinical practice with current evidence to identify areas of improvement and help guide translation of evidence-based dosages into practice. This study aimed to investigate the following questions through a medical record audit in a swallowing rehabilitation setting:

1. How is dosage of swallowing exercises recorded in clinical practice?
2. What dosages and types of swallowing exercises for stroke rehabilitation are reported in a single inpatient rehabilitation unit?

We were interested in exploring whether dosages of swallowing exercises as identified in the audit followed principles of neural plasticity and strength training specific to dysphagia. As data were collected on swallowing exercise dosages and outcome measures, we explored an additional study aim through statistical analysis. To add to the evidence base on the relation between dosage and outcomes, we explored the question:

(3) Is there a relation between swallowing exercise dosage and the likelihood of improvement in functional oral intake scale?

## Methods

### Design and Ethics

This study was conducted as a retrospective medical record audit. Ethical approval for this study was received from the local human research ethics committee (ETH12433) and site-specific governance office.

### Setting

The audit was conducted in the rehabilitation ward at a subacute rehabilitation hospital. The hospital services a multicultural population, with 60% of local residents born in an overseas country [54]. The 36-bed rehabilitation unit services a range of patients, including stroke patients. The speech pathology department works 1.5 full-time equivalent hours across all wards and outpatient clinics.

### Participants

Consecutive medical records were audited from March 2018 to September 2019. Participants were included if they met the following criteria: patients with post-stroke oral or pharyngeal dysphagia admitted to a subacute rehabilitation unit over an 18-month period who received rehabilitative swallowing exercises. Participants were included regardless of language spoken, cognitive or communication status, or reason for discharge (i.e. including patients transferred to other facilities due to medical deterioration).

Speech pathology patient goal sheets were reviewed from March 2018 to September 2019 to identify a list of patients who matched inclusion criteria. To ensure all eligible patients were found, this list was cross-referenced with a list generated from the electronic database of patients who had been coded with ‘Dysphagia’ on their electronic medical record.

### Data Collection

Data were extracted from medical records using a data abstraction spreadsheet by the lead researcher. Data were directly extracted from medical records on participant age, sex, primary language, stroke type and location, medical history, Functional Independence Measure (FIM) [55], admission and discharge dates, and discharge destination. Data were also collected on type of dysphagia according to swallowing phase (i.e. oral, pharyngeal, oropharyngeal), dysphagia severity (based on Australian Therapy Outcome Measures [56]), swallowing exercises, recorded exercise dosages and equipment used during swallowing intervention sessions, and prescription of and compliance with swallowing home exercise programs. Information from physiotherapy, occupational therapy, speech pathology, and medical progress notes was used by the lead author to score stroke severity using the Modified Rankin Score (MRS) [57], and pre- and post-oral intake using the Functional Oral Intake Scale (FOIS) [58].

To record exercise dosage, the number of swallowing exercise repetitions per session was recorded for each participant. Isotonic exercise repetitions were counted as separate repetitions (e.g. one tongue press = one exercise repetition, one head lift = one exercise repetition). Isometric exercise repetitions were counted as separate repetitions regardless of length (e.g. one 10-s jaw opening against resistance = one exercise repetition, one 60-s head lift = one exercise repetition). For swallow-specific exercises, each swallow was counted as an exercise repetition (e.g. one Mendelsohn manoeuvre = one exercise repetition).

Exercises were grouped into categories: (1) Indirect oral exercises, which targeted strength, range of motion, and coordination of muscles involved in the oral phase of swallowing, i.e. lips, tongue, and jaw; (2) Indirect pharyngeal exercises aiming to strengthen suprahyoid muscles which did not involve a swallow, e.g. Shaker head lift, chin tuck against resistance; (3) Direct exercises which involved a swallow, e.g. swallowing a bolus, Mendelsohn manoeuvre [59].

### Data Analysis

Data were collated and reported descriptively. Data on each element of the FITT exercise framework (frequency, intensity, time/duration, and type of exercise) were recorded. The overall duration of intervention was measured as the number of days from initial swallowing assessment to discharge from active swallowing intervention (not including weekends or public holidays). Frequency was recorded as the number of swallowing therapy sessions received divided by the number of weeks of intervention (e.g. 14 sessions over seven weeks = frequency of two sessions per week). The number of repetitions of each swallowing exercise per session was recorded for each participant. The mean, median, minimum, and maximum number of repetitions per session for each swallowing exercise were calculated across participants. Any measures of intensity reported in medical records were noted, such as use of surface electromyography and subjective ratings of effort. The total number of swallowing exercise repetitions divided by the overall duration of intervention was used to calculate the mean number of swallowing exercise repetitions received per day (e.g. 120 exercise repetitions over 10-day admission = 120/10 = mean daily dose of 12 exercise repetitions).

In order to evaluate trends in dosage prescription over time, post-hoc Mann Kendall tests were conducted using XLSTAT on Microsoft Excel (Microsoft Office 365 Version 2108) in conjunction with a university statistician. To reduce multiple testing and ensure adequate data points, the seven pharyngeal swallowing exercises completed by at least eight participants each were selected for statistical analysis. For each exercise, the average number of swallowing exercise repetitions completed were calculated for each session (e.g. for session 1, session 2, session 3) where three or more data points were available. Mann Kendall tests with Bonferroni corrections (significance level set at *p* = 0.007) were conducted to determine if there were any trends in the average number of swallowing exercise repetitions conducted over sessions.

Statistical analysis was conducted to explore the relation between dosage and likelihood of change in FOIS. Stepwise multivariate logistic regression analysis was conducted using IBM SPSS Statistics for Windows, Version 25 in conjunction with a university statistician. The mean number of swallowing exercise repetitions per day was used as a measure of dosage. The dependent variable was whether there was improvement in the participants’ FOIS score. Data visualization identified outliers and confounding variables that impacted on change in FOIS. Regression modelling was used to create a minimal regression model.

## Results

### Participants

Forty-two participants were included in the audit. The mean age of participants was 68.4 (SD: 13.8) years. Thirteen different languages were spoken by participants and eighteen participants (43%) required an interpreter. Six participants had an incomplete admission and were transferred to another hospital due to medical deterioration. Two of these patients were readmitted to the inpatient unit, but only their first admissions were audited. Most participants (83%) had a FOIS of 5 on admission, which represented a total oral diet with multiple consistencies requiring modification [58]. Thirty-five participants had communication comorbidities, including 26 with dysarthria or dysphonia (mostly mild), nine with aphasia, four with apraxia of speech, and four with cognitive-communication difficulties. Seven participants did not have communication comorbidities. See Table 1 for participant characteristics.

**Table 1:** Participant characteristics

Participant characteristics	Study population
Age, mean (range; SD)	68.4 (39–99; 13.8)
Sex, n; male/female	29/13
Language spoken, n (%)	
English	16 (38%)
Vietnamese	7 (16%)
Arabic	5 (11%)
Assyrian	2 (5%)
Cantonese	2 (5%)
Khmer	2 (5%)
Spanish	2 (5%)
Other	6 (14%)
Stroke type, n (%)	
Infarct	30 (71%)
Infarct with haemhorrhagic transformation	2 (5%)
Haemorrhage	9 (21%)
Not stated	1 (2%)
Stroke side, n (%)	
Left	12 (29%)
Right	27 (64%)
Bilateral	2 (5%)
Not stated	1 (2%)
Stroke location, n (%)	
Supratentorial	35 (83%)
Infratentorial	6 (14%)
Mixed	1 (2%)
Functional Oral Intake Scale at admission, n (%)	
1	3 (7%)
2	0 (0%)
3	0 (0%)
4	0 (0%)
5	35 (83%)
6	4 (10%)
7	0 (0%)
Functional Independence Measure, mean (range; SD)	71.0 (33–111; 18.2)
Median (variance)	73.5 (30)
Number of days between stroke onset and admission to rehabilitation ward, mean (range; SD)	22.5 (7–72; 14.8)
Median (variance)	19 (19)
Length of stay in rehabilitation ward, mean (range; SD)	27 (8–91; 18.2)
Median (variance)	21 (16)

*n* number, *SD* Standard Deviation

### Reporting of Swallowing Exercise Dosages

Several aspects of swallowing exercise dosage were found to be routinely reported in medical records. These included exercise name/type, number of exercise repetitions per session, dates and frequency of sessions, and initial and final sessions (from which intervention duration could be calculated). Intensity of swallowing exercises was not consistently reported. A rating out of five was sometimes used to rate perceived strength, range-of-motion, or speed for indirect oral exercises. When surface electromyography or oral exercises with resistance were used, the average or maximum strength rating was recorded, but number of exercise repetitions was not. For all other exercises, there was no consistent (or available) measure of exercise intensity or task difficulty. The duration of intervention sessions and length of active versus inactive therapy time were not reported. All medical records except for one reported if home exercise programs were provided. However, the content of home exercise programs was only reported in 12 medical records (29%) and patient adherence to home exercise programs was reported in 16 records (38%).

### Dosages of Swallowing Exercises

Sixteen different swallowing exercises were prescribed to participants. The most commonly prescribed exercises were chin tuck against resistance, lip and tongue exercises, and rotary chews. Overall, 39 patients were prescribed indirect oral exercises, 23 were prescribed indirect pharyngeal exercises, and 23 were prescribed swallow-specific exercises in their intervention programs. Patients received a median of 18.8 (IQR: 24.3) indirect oral swallowing exercise repetitions per session (mean: 26.9, SD: 24.9), median of 42.1 (IQR: 21.1) indirect pharyngeal swallowing exercise repetitions per session (mean: 39.4, SD: 13.0), and median of 2.1 (IQR: 3.3) direct swallow-specific exercise repetitions per session (mean: 3.1, SD: 2.8). Overall, patients received a median of 44.5 (IQR: 39.6) swallowing exercise repetitions per session (mean: 48.3, SD: 33.3).

Patients were seen for a median of 2.4 sessions per week (IQR: 1.7), mean: 2.6 sessions per week (SD: 1.3). Median intervention duration was 21 days, or 3 weeks (IQR: 16) and ranged from eight to 91 days (mean: 27 days, SD: 18.2). Patients were seen for a median of 8.5 swallowing intervention sessions (IQR: 8; range: 1–57; mean: 10.8, SD: 11.4). See Table 2 for individual swallowing exercises and dosages.

**Table 2:** Swallowing exercises and dosages reported in medical records

Swallowing exercise	No. of participants who received the exercise	Mean reps per session (SD; range)	Mode reps per session	Hold Y/N? If Y: (proportion of participants), duration of hold (range)
*Indirect oral exercises*				
Jaw exercises ± sEMG	6	2.7 (1.6; 1–6)	3	Y (2/6), 5–30 s
Lip seal/strength exercises ± resistance	22	4.7 (6.4; 0–45)	3	Y (15/22), 3–30 s
Lip range-of-movement exercises	12	24.5 (21.9; 3–90)	10	N
Tongue exercises ± swab/bolus or resistance	30	13.1 (14.8; 0–120)	5	N
Rotary chews	31	33.9 (24.9; 3–150)	30	N
Other base of tongue exercises	1	2.2 (1.8; 1–5)	5	Y (1/1), gargle 30 s; yawn 5 s
*Indirect pharyngeal exercises*				
CTAR (isometric) vs towel/ball	23	2.6 (0.8; 0–6)	3	Y (23/23), 10–140 s
CTAR (isotonic) vs towel/ball	23	50.8 (28.4; 4–180)	30	N
Shaker (isometric)	9	2.6 (0.9; 1–4)	3	Y (9/9), 10–75 s
Shaker (isotonic)	9	33.8 (19.0; 10–90)	30	N
Max jaw opening vs resistance	2	5.3 (0.6; 5–6)	5	Y (2/2), 10 s
Falsetto “ee”	3	4.5 (3.4; 0–10)	5	N
*Direct pharyngeal exercises*				
Effortful swallow ± sEMG	16	3.6 (3.5; 0–30)	3	N
Mendelsoh*n* ± sEMG	11	1.8 (1.9; 0–10)	1, 3	Y (not stated)
Supraglottic swallow	3	3.1 (1.6; 1–5)	5	N
Masako	9	2.5 (2.7; 0–18)	3	N
Chew and swallow bolus	5	4.0 (1.9; 1–7)	3	N
Swallow post stimulation	3	6.5 (3.9; 0–12)	10	N

*CTAR* chin tuck against resistance, *N* no, *No*. number, *sec* seconds, *sEMG* surface electromyography, *vs* versus, + / − with or without, *Y* yes

Patients received a median of 12.3 (IQR: 25.8) total swallowing exercise repetitions per day (mean: 18.2, SD: 16.6). Patients completed a median of 6.2 (IQR: 9.8) indirect oral swallowing exercise repetitions per day (mean: 8.7, SD: 9.3), 14.7 (IQR: 15.1) indirect pharyngeal swallowing exercise repetitions per day (mean: 17.2, SD: 9.5), and 0.8 (IQR: 1.0) direct swallow-specific exercise repetitions per day (mean: 1.4, SD: 1.3).

**Fig. 1:**
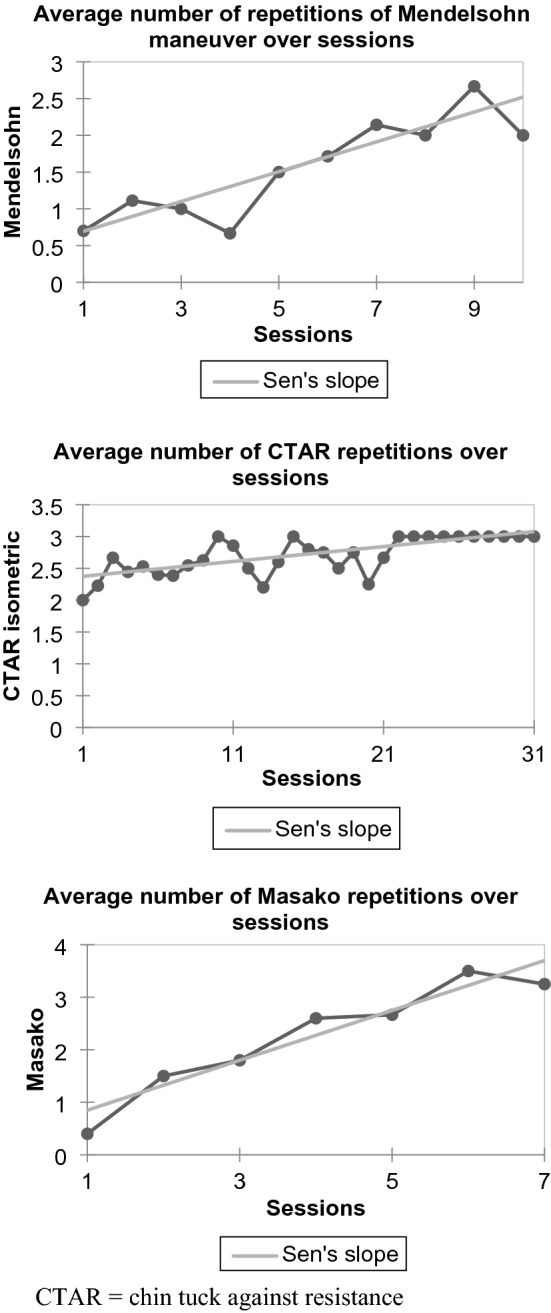
Average number of swallowing exercise repetitions over sessions

We evaluated dosage prescription over time. The amount or difficulty of swallowing exercises tended to increase for each participant over the course of their intervention. This occurred through increasing number of repetitions, length of isometric holds, or resistance levels (e.g. progressing from 30 to 60 exercise repetitions or progressing from tongue presses to tongue presses against resistance). Mann Kendall tests (with Bonferroni corrections) conducted with the seven most commonly used pharyngeal swallowing exercises showed mixed results. A significant positive trend in the number of exercise repetitions over sessions was found for Mendelsohn manoeuvre (τ = 0.67, S = 30, *p* = 0.007), Masako manoeuvre (τ = 0.91, S = 19, *p* = 0.003) and isometric chin tuck against resistance (τ = 0.57, S = 244, *p* < 0.0001) (see Fig. 1 for Sen’s slopes showing average swallowing exercise repetitions over sessions). This indicates that the average number of swallowing exercise repetitions increased as sessions progressed. However, no other significant trends were found for the other four swallowing exercises (isometric and isokinetic Shaker, isokinetic chin tuck against resistance, or effortful swallow).

### Relation Between Swallowing Exercise Dosage and Likelihood of Improvement in Oral Intake

Data on patients’ change in FOIS were positively skewed with 18 patients demonstrating no change in FOIS, and 23 patients demonstrating improvement in FOIS to different extents. As a result, the swallowing outcome measure was expressed as a dichotomous variable of improvement in FOIS versus no improvement. Patients who improved in their FOIS scores had a lower mean age and received a higher number of swallowing exercise repetitions per session or per day compared to patients whose FOIS scores did not improve (see Table 3). Thirty-eight per cent of patients whose FOIS scores improved required an interpreter, while 50% of patients whose FOIS scores did not improve required an interpreter. Regression modelling indicated that the only statistically relevant factor was age. None of the other factors were statistically significant.

**Table 3:** Patient characteristics and number of swallowing exercise repetitions

	Improvement in FOIS (*n* = 24)	No improvement in FOIS (*n* = 18)
Age, mean (SD)	63.6 (14.1)	74.8 (11.7)
Sex, n; male/female	17/7	11/7
Interpreter required, n (%)	9 (37.5%)	9 (50%)
Functional Independence Measure at admission, mean (SD)	72.2 (17.1)	69.4 (19.9)
Number of days between stroke onset and admission to rehabilitation ward, mean (SD)	22.7 (13.4)	22.3 (16.9)
FOIS at admission, median (IQR)	4.7 (1.1)	5.0 (1.1)
Total number of swallowing exercise repetitions per session		
Mean (SD)	54.8 (29.4)	39.6 (36.9)
Median (IQR)	49.8 (41.7)	33.8 (40.0)
Total number of swallowing exercise repetitions per day		
Mean (SD)	21.1 (15.0)	14.4 (18.2)
Median (IQR)	18.2 (22.9)	6.6 (17.2)

*FOIS* functional oral intake scale, *IQR* interquartile range, *n* number, *SD* standard deviation

Visual inspection of data scatterplots identified a deviant outlier. This participant had profound dysphagia (FOIS 1) without any improvement despite intervention and was removed from regression analysis to avoid bias and skewing of data. Age and mean number of swallowing exercise repetitions per day were then used in a stepwise logistic regression model. Results indicated that age negatively impacted on whether there was change in FOIS to a small extent (B = −0.015, *p* = 0.007), but number of exercise repetitions per day did not impact on FOIS change (B = 0.004, *p* = 0.363) (see Table 4).

**Table 4:** Multivariate logistic regression coefficients

Model		Unstandardized coefficients		Standardized coefficients	t	*p* value
		B	Standard error	Beta		
1	(Constant)	1.582	.356		4.442	.000
	Age patient age	−.015	.005	−.416	−2.857	.007
2	(Constant)	1.474	.376		3.899	.000
	Age Patient age	−.014	.005	−.402	−2.740	.009
	Total Reps Per Day	.004	.004	.135	.920	.363

Dependent Variable: Change in FOIS (binary)

*B* Unstandardized beta, *t* t statistic, *Total Reps Per Day* total number of swallowing exercise repetitions per day

## Discussion

This study investigated how and what dosages of swallowing exercises were reported in clinical practice and additionally explored the relation between exercise dosage and change in functional oral intake status. We found that not all aspects of dosage were routinely reported in medical records, and that dosage prescription did not consistently adhere to evidence-based principles. The dosage of swallowing intervention was generally low compared to stroke rehabilitation studies [49, 50]. Indirect swallowing exercises were conducted more than direct swallowing exercises, and strength-training principles were incorporated but not consistently applied. Age impacted on whether functional oral intake status improved, but number of swallowing exercise repetitions per session did not. Overall, this study provides useful information on common types and dosages of swallowing exercises in clinical practice as a baseline for clinicians and researchers. Below we discuss how findings compare with evidence-based principles in stroke rehabilitation, potential reasons, and areas for further consideration.

This pragmatic study is the first of its kind to report on the dosages of swallowing exercises used in practice. The medical record audit design was used to capture a picture of typical dosage reporting and implementation in a clinical setting. Findings revealed that dosage was inconsistently reported, and swallowing exercises and dosages varied significantly. Frequency, intervention duration, and exercise type were routinely reported in medical records. However, intervention intensity, therapy time, and the amount and content of independent practice were not consistently reported. There was variation in the type of swallowing exercises and dosages used. Sixteen different swallowing exercises were used, which included indirect oral and pharyngeal, and direct swallowing exercises. The number of swallowing exercise repetitions and length of isometric exercise holds varied, with wide ranges observed. The inconsistent reporting and variation of dosages highlight the need for consistent dosage definition and guidelines around reporting of swallowing exercise dosages in clinical practice.

The findings from this study can be viewed in light of key dosage principles in stroke rehabilitation, particularly amount of intervention, specificity, and strength-based exercise training. Firstly, the amount of exercise reported in this medical record audit is lower compared to exercise dosages reported in research. The average frequency of intervention reported in this study (two–three days per week) was less than swallowing intervention studies (on average, five days per week) [49]. However, the number of exercise repetitions per session and overall intervention duration were largely similar to swallowing intervention studies, given heterogeneity in research and clinical settings [49, 50]. Exercise dosages in clinical practice are expected to be lower than research studies due to real-life factors such as patient compliance, clinician availability, and competing rehabilitation goals [60]. This study illustrates the dosages of swallowing exercises that can be achieved in a real-life clinical setting, including with a culturally and linguistically diverse patient population. Given the importance of high dose intervention in stroke rehabilitation [23, 45], the gap between dosages used in research and dosages used by clinicians highlights the need to increase clinical swallowing exercise dosages.

Secondly, the swallowing exercise patterns reported in this study did not adhere to the neural plasticity principle of specificity [20]. Direct swallowing exercises were provided significantly less than indirect swallowing exercises. Swallow-specific exercises can be understandably more challenging for patients with swallowing difficulties to complete. Indirect swallowing exercises play an important role in strengthening swallowing-related muscles, particularly when patients are unable to swallow and have been shown to be effective in improving swallow function [61, 62]. However, strong evidence in limb training reports the benefits of task-specific practice [20, 30]. Swallowing studies demonstrate positive outcomes with swallow-specific intervention programs, such as McNeill Dysphagia Therapy Program [63, 64], swallow accuracy training [65], and thermal-tactile stimulation [66]. This evidence and the findings of this audit suggest the benefits of increasing functional swallowing practice in intervention.

Thirdly, swallowing exercise dosages only partially followed strength-training exercise principles. The majority of swallowing exercises reported in this audit were strengthening exercises. However, key principles of strength training – including progressive overload and resistance training – were not systematically followed [34]. Exercises can be progressed through increasing resistance, number of repetitions per set, or frequency of training [67]. The number of swallowing exercise repetitions increased over time, with significant positive trends for some exercises (isometric CTAR, Mendelsohn and Masako). However, results were mixed, with no clear trends in dosage progression for other swallowing exercises. While there is insufficient data to explain why only some exercises increased in dosage over time, it is likely that patient tolerance, clinician priorities, time limitations, and data reporting impacted on this data. Some exercises included resistance, including lip and tongue exercises performed against resistance from a tongue depressor, swab or hand, and chin tuck and jaw opening against resistance exercises. However, the forms of resistance reported in this audit could not systematically be increased or altered over time. Studies are emerging on potential ways to incorporate systematic progressive resistance into swallowing exercises (see [68–70] for examples). However, further research is needed to identify how to optimally apply strength principles to swallowing exercises, and how to set and progress swallowing exercise repetition targets over time.

There are several potential reasons why exercise dosages in this audit did not match dosages or dosage principles in research. Competing priorities and time limitations impact clinicians’ ability to access and navigate research [71]. The plethora of studies related to dosage and swallowing intervention need to be simplified into accessible practice guidelines to facilitate application of research into practice [72]. Differences between research participants and real-life patients also limit the implementation of dosage principles into practice. Around 80% of patients in this study had communication difficulties and over 60% were from non-English-speaking backgrounds – two groups underrepresented in stroke research [73–75]. Reduced communication and English-speaking ability can be barriers to patients’ participation in stroke rehabilitation [76–78] and reduce the amount of intervention received. Finally, funding and staffing levels impact clinician availability. The limited staffing at the audit site (1.5FTE across all wards) likely impacted the frequency and duration of swallowing intervention. Inconsistency, variability, and low staffing of speech pathology services have been demonstrated in service provision surveys in wards such as intensive care units and spinal rehabilitation [79, 80]. More information is needed on barriers and facilitators to dosage prescription and implementation in swallowing intervention to facilitate translation of evidence-based dosage principles into clinical practice.

This study further investigated the relation between patient characteristics and likelihood of improvement in FOIS. Age was negatively correlated with improvement in functional swallowing status, i.e. the younger the patient, the more likely that their FOIS improved by discharge from rehabilitation. This is in line with research demonstrating younger age as a predictor of recovery of oral intake in acute and rehabilitation settings [81–83]. Most studies have investigated the impact of age on returning to oral intake (rather than long-term non-oral intake) [81–83]. Our study investigated a caseload with less severe dysphagia and found a similar impact of age on returning to premorbid diet and fluids.

The finding of no relation between number of swallowing exercise repetitions and improvement in swallowing outcome contrasts with other studies. Two retrospective studies found that an increased amount of swallowing intervention had a positive impact on outcomes. Nakazora et al. [84] conducted a retrospective cohort study comparing weekday speech therapy (five days per week) with daily speech therapy (seven days per week) for patients with dysphagia due to acute stroke. Multiple linear regression with 452 patients found that daily swallowing therapy was associated with reduced number of days until oral intake commenced. Gittins et al. [85] conducted a large secondary analysis of stroke audit data and allied health therapy. In 59,537 adults, more speech therapy (calculated as amount of therapy time) was associated with shorter length of stay, lower mortality, disability, and institutionalization at discharge. Both studies found that an increased amount of speech therapy was associated with faster recovery of oral intake or improved prognosis.

The differences in findings between our study and the two papers described may be due to sample size and selected outcome measures. A limitation of our study was the small sample size, which reduced power and may have contributed to the finding of no statistical relation between mean daily number of swallowing exercise repetitions and improvement in FOIS. Further research is needed to explore if the trends observed in this study – with number of swallowing exercise repetitions and use of interpreters – persist and are significant in a larger sample size. Due to a smaller sample size, there was also less variation in dysphagia severity and swallowing exercise dosages, and the threshold needed to observe effects of increased intervention may not have been met [26]. Further, all studies used different outcome measures, which could explain differences in results. Change in FOIS may not have been sensitive enough to demonstrate differences in our primarily mild-to-moderate dysphagia caseload. Finally, our finding of inconsistent reporting of exercise dosage in medical records highlights that the retrospective audit may not have reflected true practice. Prospective intervention studies with controlled variables are needed to accurately investigate the relation between exercise dosage and swallowing outcomes.

A further challenge in this study was the difficulty quantifying exercise dosage. Counting exercise repetitions is considered a better measure of exercise dosage than amount of scheduled or actual therapy time [22, 86]. However, counting exercise repetitions does not account for differences in isometric versus isotonic, or indirect versus direct exercise types. Further, the measure of exercise dosage in this study (mean number of exercise repetitions per day) did not account for dosing rates. For example, a patient who completed 60 repetitions in two sessions over ten days would have the same mean daily dose as a patient who completed six repetitions per day over 20 days, despite different dosing rates. However, alternative measures are also inadequate to capture exercise dosage. For example, number of repetitions per session does not account for frequency of sessions or overall intervention duration, and frequency of sessions does not account for number of exercise repetitions or duration. As such, mean number of exercise repetitions per day was selected as a broad measure of dosage, in line with other studies [87]. However, these issues highlight the complexity of measuring exercise dosage.

Our findings indicate the need for a better understanding of dosage and evidence-based dosage principles in post-stroke dysphagia. A consistent definition of dosage is needed to improve measurement and reporting of dosage in clinical care to facilitate handover and replication, monitor goal achievement, guide task progression, and allow comparison of results [86, 88, 89]. Despite this, lack of clear guidelines for defining and reporting dosage results in inconsistent descriptions of dosage in intervention studies [49, 50, 89], similar to the inconsistent reporting of dosage found in medical records in this audit. Various frameworks for defining and reporting dosage exist to facilitate consistent dosage reporting [90–92]. One definition of dosage, borrowed from exercise physiology, is the FITT model used in this study [3]. This easy to remember framework can guide consideration of the components of frequency, intensity, time, and type when prescribing and reporting on swallowing exercise dosage. However, further work is needed to define dosage and establish clinical guidelines for reporting on dosage in research and clinical contexts.

There are several implications for clinical practice. Clinicians should be mindful of dosage when prescribing and planning swallowing intervention. Stroke research identifies dosage principles that can apply to swallowing intervention, particularly increasing amount of intervention, specificity of practice, and use of strength-training principles. However, this audit revealed that swallowing intervention dosage in practice is lower than in research studies, may not be swallow-specific or consistently follow progressive strengthening principles. In order to increase dosage and adherence, clinicians can use technology such as websites and apps [93, 94], home exercise programs, and behavioural strategies such as written instructions, setting behavioural goals, and social support [95,96]. Strategies for increasing direct swallowing practice include the use of real boluses, biofeedback, and swallow stimulation [63, 65, 66]. Finally, clinicians should increase the intensity or resistance of swallowing exercises as patients improve, through systematically increasing the number of repetitions, length of exercise holds, or use of devices to apply or measure resistance [97–99] such as EMST [100], IOPI [68], and sEMG [101]. These preliminary suggestions can aid in improving evidence-based dosages of swallowing exercise repetitions, but further work is needed to clarify how dosage principles apply to swallowing intervention and to facilitate translation of evidence-based knowledge into everyday clinical practice.

## Data Availability

The data that support the findings of this study are available from the corresponding author upon reasonable request.
